# Synthesis, Biological Evaluation and Molecular Modeling Studies of Naphthoquinone Sulfonamides and Sulfonate Ester Derivatives as P2X7 Inhibitors

**DOI:** 10.3390/molecules28020590

**Published:** 2023-01-06

**Authors:** Paulo Anastácio Furtado Pacheco, Daniel Tadeu Gomes Gonzaga, Natalia Lidmar von Ranke, Carlos Rangel Rodrigues, David Rodrigues da Rocha, Fernando de Carvalho da Silva, Vitor Francisco Ferreira, Robson Xavier Faria

**Affiliations:** 1 Department of Organic Chemistry, Institute of Chemistry, Federal Fluminense University, Niterói 24020-141, Brazil; 2 Departament of Pharmacy, West Zone Campus, State University of Rio de Janeiro, Rio de Janeiro 23070-200, Brazil; 3 Department of Pharmaceuticals and Medicines, Faculty of Pharmacy, Federal University of Rio de Janeiro, Rio de Janeiro 21941-170, Brazil; 4 Evaluation and Promotion of the Ambiental Health Laboratory, Oswaldo Cruz Institute, Oswaldo Cruz Foundation, Rio de Janeiro 21040-360, Brazil; 5 Postgraduate Program in Sciences and Biotechnology, Institute of Biology, Federal Fluminense University, Niterói 24210-130, Brazil

**Keywords:** naphthoquinones, sulfonamides, heterocycles, biomass, ATP, inflammation

## Abstract

ATP acts in the extracellular environment as an important signal, activating a family of receptors called purinergic receptors. In recent years, interest in the potential therapeutics of purinergic components, including agonists and antagonists of receptors, has increased. Currently, many observations have indicated that ATP acts as an important mediator of inflammatory responses and, when found in high concentrations in the extracellular space, is related to the activation of the P2X7 purinergic receptor. In this sense, the search for new inhibitors for this receptor has attracted a great deal of attention in recent years. Sulfonamide derivatives have been reported to be potent inhibitors of P2X receptors. In this study, ten naphthoquinone sulfonamide derivatives and five naphthoquinone sulfonate ester derivatives were tested for their inhibitory activity on the P2X7 receptor expressed in peritoneal macrophages. Some compounds showed promising results, displaying IC_50_ values lower than that of A740003. Molecular docking and dynamic studies also indicated that the active compounds bind to an allosteric site on P2X7R. The binding free energy indicates that sulfonamides have an affinity for the P2X7 receptor similar to A740003. Therefore, the compounds studied herein present potential P2X7R inhibition.

## 1. Introduction

Purinergic receptors are transmembrane receptors widely expressed in mammalian tissues. These receptors are involved in several physiological and pathological processes, such as cell proliferation, migration and differentiation, cytokine production, pain, apoptosis, and tumorigenesis [1]. Initially, they were classified into two subgroups depending on their responsiveness to extracellular adenosine (P1 receptors) or purine/pyrimidine nucleotides (P2 receptors) [2,3]. P1 receptors are G protein protein-coupled receptors (GPCRs) that differ in the type of associated G protein. Four P1 receptor subtypes have been identified in vertebrate tissues (A_1_, A_2A_, A_2B_ and A_3_) [4]. The P2 receptor family is further subdivided into P2X and P2Y based on function and structural composition [5,6]. While P2Y receptors are also GPCRs, activated by ATP and purine/pyrimidine nucleotides, P2X receptors are ATP-gated ion channels with seven subtypes identified in mammalian cells (P2X1R–P2X7R) [7]. There has been increasing interest in the subtype P2X7R since it differs in some aspects from the other P2XRs. From a structural point of view, it has a long intracellular carboxy-terminal tail [8]. From the point of view of its physiological activation, its affinity for the ATP molecule is much lower, requiring the use of higher concentrations for activation (EC_50_ >> 100 µM) [9]. In cells of the immune system, the response to the stimulus caused by ATP, which may come from the death of cells in its surroundings, provides the opening of the intrinsic cation channel for Na^+^, K^+^ and Ca^2+^ ions, which can promote changes in enzymatic activity and intracellular tissue triggering the release of pro-inflammatory cytokines, such as interleukin-1β (IL-1β), through a cascading chain of events [10]. These cytokines can act again on pannexin-1 (Panx-1) or connexin hemichannel (Cx43) channels, modulating the inflammatory T-cell response and macrophage migration [11]. These immune response activation mechanisms place P2X7R at the center of many inflammatory, autoimmune, and neuropathic pain [12], cancer [13], cardiovascular disease [14], and neurological disorders [15]. It is believed that control over its regulation can result in novel treatments for several diseases. In the last twenty years, some pharmaceutical companies have developed a series of substances for efficacy testing for P2X7R inhibition in models of peripheral diseases, such as osteoarthritis, rheumatoid arthritis, and Crohn’s disease [16]. In the last decade, there has also been a relevant increase in studies on the role of P2X7R in the CNS, where the target is neuroinflammation, neuropathic pain and neurodegenerative diseases [17,18]. There is a new trend in studies on this receptor aiming to build a better understanding of its participation in the pathophysiology of numerous diseases. These efforts have greatly aided in the development of numerous classes of substances capable of actively interfering with the regulation of the P2X7R response with therapeutic potential to act in the treatment of several diseases [11,19]. The fact that these molecules are not used in therapy lies in their low selectivity (they can inhibit other P2 receptors), in addition to their physicochemical and pharmacokinetic characteristics [12].

In recent years, our research group has focused on the identification of novel and more selective chemotypes of P2X7 receptor inhibitors. In particular, we reported several natural and synthetic compounds with potent antagonist activity toward P2X7R and ATP-induced inflammatory responses [20,21,22,23,24,25,26]. Recently, we reported the synthesis and biological evaluation of a group of naphthoquinones sulfonamide and sulfonate ester derivatives against Chikungunya virus (CHIKV). Since inflammatory responses are directly related to CHIKV disease pathology, and motivated by the promising results of these compounds, we evaluated the inhibitory effect against P2X7R [27,28].

## 2. Results and Discussion

### 2.1. Chemistry

**Figure 1:**
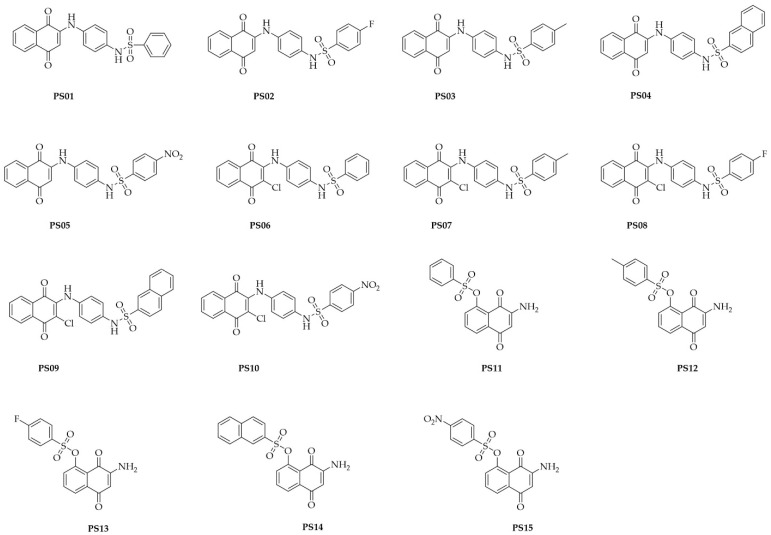
Chemical structures of naphthoquinone sulfonamide and sulfonate ester derivatives tested in this study.

The compounds were synthetized as reported in a previous study [28]. The chemical structures of the assessed compounds are shown in Figure 1.

### 2.2. Cytotoxicity Assay

**Figure 2:**
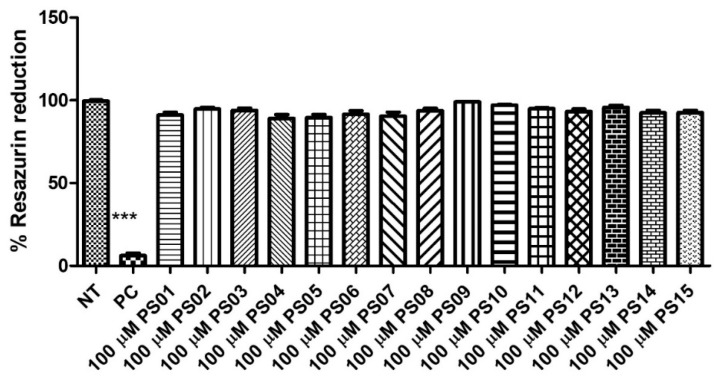
Quantification of cellular metabolic activity after treatment with the PS01–15 series employing the resazurin reduction capacity. Cells were treated with 10 µM of the substances and 0.5% TX−100 in the positive control (PC). Nontreated cells correspond to the negative control (NT). Comparison of NT vs. treatments. The error bar is representative of 3 experiments in triplicate on 3 different days. ANOVA (***) *p* < 0.0001.

Initially, we evaluated the toxicity of 15 naphthoquinone derivatives (PS01−15) in mouse peritoneal macrophages continuously exposed for 24 h. All compounds showed no significant cytotoxicity (Figure 2) when compared with untreated cells (negative control). As expected, the detergent Triton X−100 impaired the resazurin reduction in treated cells. This absence of a PS series effect on cell metabolism motivated further investigations into their effect on P2X7 antagonism [29,30,31].

### 2.3. Dye Uptake Assay

**Figure 3:**
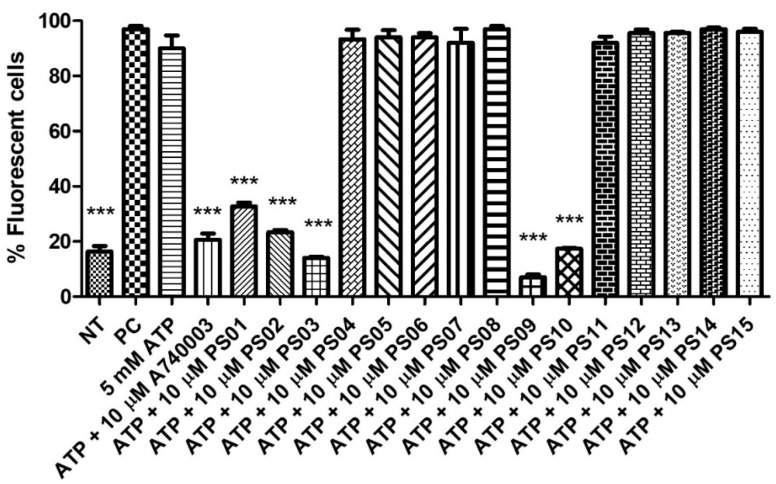
P2X7 receptor−induced PI uptake. Peritoneal macrophages were pretreated for 10 min with sulfonamides at 10 µM and subsequently stimulated with 5 mM ATP for 15 min. As an inhibition control, the cells were treated with A740003 at 10 µM for 10 min. The positive control (PC) was 0.5% Triton−X 100. Nontreated cells correspond to the negative control (NT). The bar error is representative of 3 experiments in triplicate on 3 different days. ANOVA (***) *p* < 0.0001.

Stimulation of peritoneal macrophages with ATP at millimolar concentrations induces the opening of pores permeable to fluorescent dyes, such as propidium iodide [32,33] Therefore, we preincubated these cells with a sulfonamide series for 5 min before ATP addition. Treatment with ATP induced submaximal PI uptake compared with the positive control, and A740003 inhibited the ATP effect. The substances PS04, PS05, PS06, PS07, PS08, PS11, PS12, PS13, PS14 and PS15 did not show inhibitory effects (at a concentration of 10 µM). PS01, PS02, PS03, PS09, and PS10 reduced the ATP effect by more than 50% (Figure 3).

**Figure 4:**
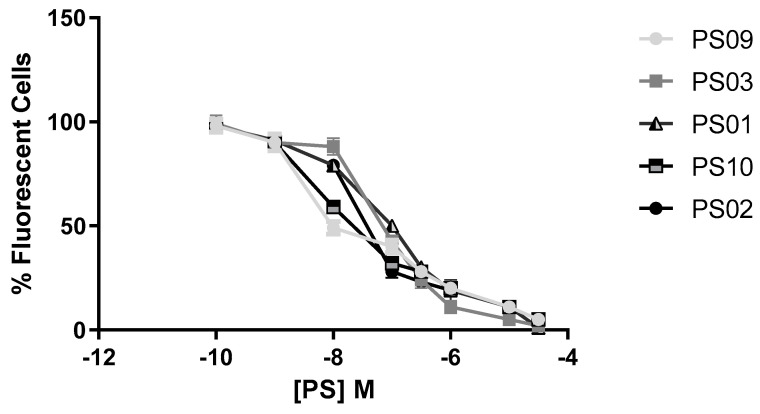
Dose−response curve for the sulfonamides. Peritoneal macrophages were pretreated for 10 min with sulfonamides at crescent concentrations and subsequently stimulated with 5 mM ATP for 15 min. As an inhibition control, the cells were treated with A740003 at 10 µM for 10 min (data not shown). The error bar is representative of 3 experiments in triplicate on 3 different days. M – drug concentration.

Sulfonamides with inhibitory activity higher than 50% (PS01, PS02, PS03, PS09, and PS10, see Figure 3) on ATP-induced PI uptake were selected and evaluated at different concentrations. We observed a dose-dependent inhibition of ATP-induced PI uptake for all five compounds (Figure 4). We estimated IC_50_ values of 1 ± 0.1 µM, 0.3 ± 0.003 µM, 0.07 ± 0.003 µM, 0.008 ± 0.0001 µM, and 0.01 ± 0.002 µM for PS01, PS02, PS03, PS09, and PS10, respectively. All selected sulfonamides exhibited IC_50_ values better than BBG, a well-known P2X7 receptor inhibitor [25,34]. Compounds PS03, PS09, and PS10 showed IC_50_ values lower than that of A740003.

**Figure 5:**
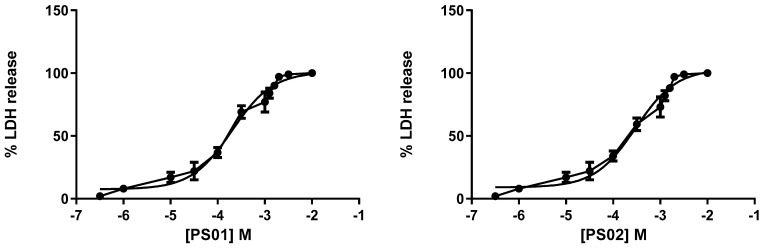

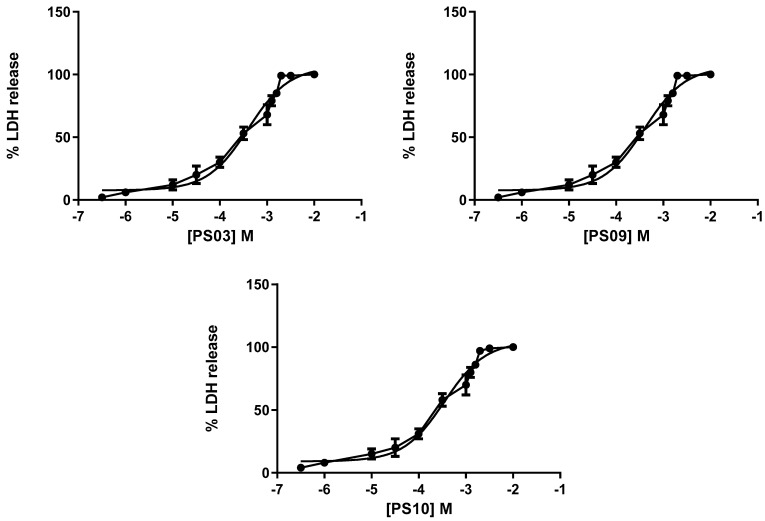
Dose-response curve for the sulfonamides. Peritoneal macrophages were pretreated for 24 h with sulfonamides at crescent concentrations. The error bar is representative of 3 experiments in triplicate on 3 different days. M—drug concentration.

Although the selected sulfonamides showed negligible alterations in cellular metabolism (see Figure 2), we tested their toxicity with increasing concentrations in mouse peritoneal cells by measuring lactate dehydrogenase (LDH) activity. Once again, the CC_50_ values for all compounds indicated no toxicity [35,36]. We determined IC_50_ values of 201 ± 11 µM, 298 ± 16 µM, 322 ± 20 µM, 401 ± 18 µM, and 351 ± 21 µM for PS01, PS02, PS03, PS09, and PS10, respectively (Figure 5).

Another hallmark of P2X7R activation in the inflammatory response is IL−1β release after ATP stimulus. Thus, we treated human THP-1 cells and mouse peritoneal macrophages with LPS for 4 h and added ATP for 30 min to increase IL−1β release. Pretreatment with BBG, A740003 and all selected sulfonamides reversed this effect. Interestingly, PS01, PS02, and PS03 were able to inhibit P2X7R-mediated IL−1β release at low micromolar concentrations (Table 1). This inhibition was higher than BBG inhibition and worse than A740003. PS09 and PS10 exhibited higher inhibitory activity (Table 1). When compared with the ATP−induced pore formation assay, we observed an inversion of potency inhibition order. The distinct intracellular signaling pathways associated with the P2X7R pore and P2X7R/inflammasome/IL−1β pathways might justify this discrepancy [33].

**Figure 6:**
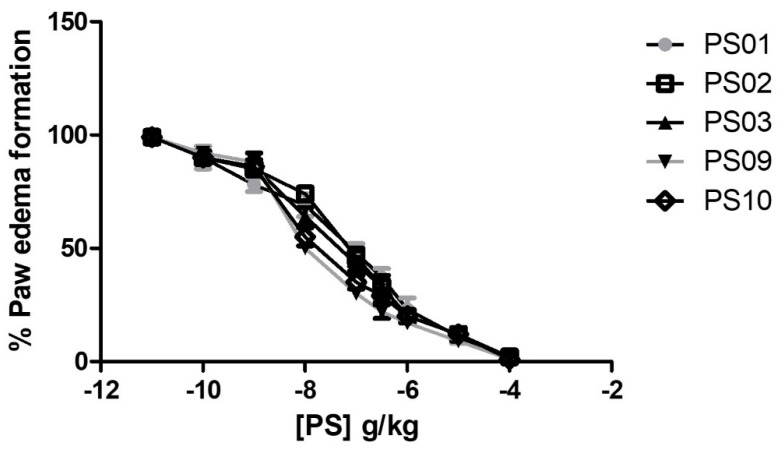
Anti−edematogenic effects of sulfonamides on ATP-induced paw edema in mice. Groups with five Swiss Webster mice treated with ATP (intra-plantar) were preincubated for 30 min with increasing doses of sulfonamides. All sulfonamide data were compared with crescent BBG doses (data not shown). Paw edema was measured at 30 min after ATP application. These results represent three distinct days with five animals for each group.

To evaluate the anti-inflammatory effects of the selected sulfonamides in vivo, we performed an ATP−induced edema formation assay. Treatment with all selected sulfonamides inhibited ATP−induced edema formation with ID50 values of 128 ± 16 ng/kg, 93 ± 8 ng/kg, 67 ± 5 ng/kg, 9.8 ± 0.7 ng/kg, and 13.6 ± 16 ng/kg for PS01, PS02, PS03, PS09, and PS10, respectively (Figure 6). Interestingly, the anti-inflammatory effect of selected sulfonamides revealed PS09 and PS10 as the most promising P2X7R antagonists, although all derivatives exhibited potent inhibitory effects.

### 2.4. Molecular Docking and Dynamics

**Figure 7:**
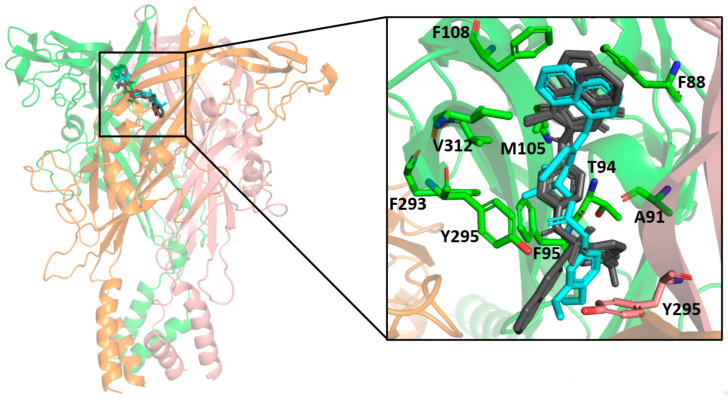
Superposition of the docked sulfonamide compounds into the P2X7 allosteric site. P2X7R is depicted in the cartoon, and each chain is represented in a different color. The sulfonamide ligands are depicted in gray, and the A740003 ligand is depicted in cyan. The main interacting residues are depicted in an enlarged view. The A740003 structure conformation was retrieved from PDB (ID: 5U1U).

The molecular docking results indicated a similar binding conformation for the sulfonamide compounds into the P2X7 allosteric pocket. The 1,4-naphthoquinone moiety of the sulfonamide compounds superposed well with the quinolinyl moiety of A740003 from the crystal structure (PDB: 5U1U). Thus, both moieties showed hydrophobic interactions with the residues Phe108, Val312, Met105, and Phe88. In addition, the sulfonamide compounds also presented interactions with residues located deep within the hydrophobic cavity, such as Tyr295, Phe95, Phe293, and Thr94. These interactions were also reported in the co-crystallization work of Karasawa and Kawate [37]. Furthermore, the 1,4-naphthoquinone core was already suggested as a pharmacophore group for P2X7 allosteric inhibitors by our previous work [38]. Figure 7 presents the superposition of the sulfonamide compounds docked into the allosteric site, as well as the A740003 ligand.

**Figure 8:**
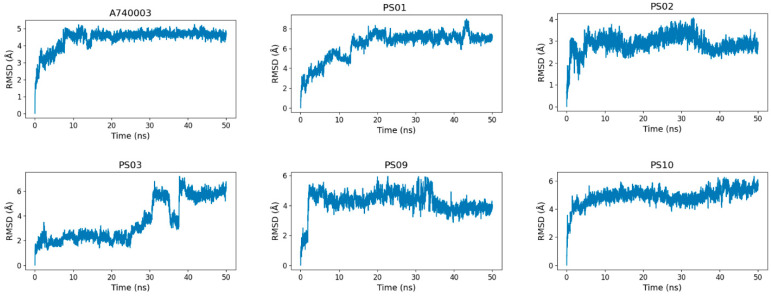
RMSD (root-mean-square deviation) variation of the ligands (A740003, PS01, PS02, PS03, PS09, and PS10) in relation to the P2X7 receptor during 50 ns of molecular dynamics simulation.

To assess the sulfonamide compound stability and binding free energy into the P2X7 allosteric site, we performed molecular dynamics simulations of 50 ns for P2X7 in complex with each of the top five sulfonamides and, for comparison, the A740003 ligand. All 6 ligands (A740003, PS01, PS02, PS03, PS09, and PS10) complexed with the P2X7 receptor presented a stable trajectory conformation (Supplementary Materials, Figure S1). The ligand RMSD variation in relation to the P2X7 receptor indicated that the ligands were accommodated in the pocket at the beginning of the simulation and remained stable until the end (Figure 8). Although compound PS03 accommodated approximately 40–50 ns, it also remained stable in the allosteric site until the end of the simulation.

After completing the 50 ns of simulation, all sulfonamide compounds slightly moved toward the inner of the P2X7 ion channel pore, becoming more buried into the allosteirc site. Therefore, the sufonamide compounds had a high affinity for this cavity. By analyzing the last frame from the molecular dynamics for each complex, we observed that the 1,4-naphthoquinone core of the sulfonamide compounds interacted mainly with the hydrophobic lateral chain of Val312, Ile310, Met105, and Phe88. In addition, PS01, PS02, and PS03 established a hydrogen bond between their secondary amine and the oxygen from Ala91 or Thr94.

**Figure 9:**
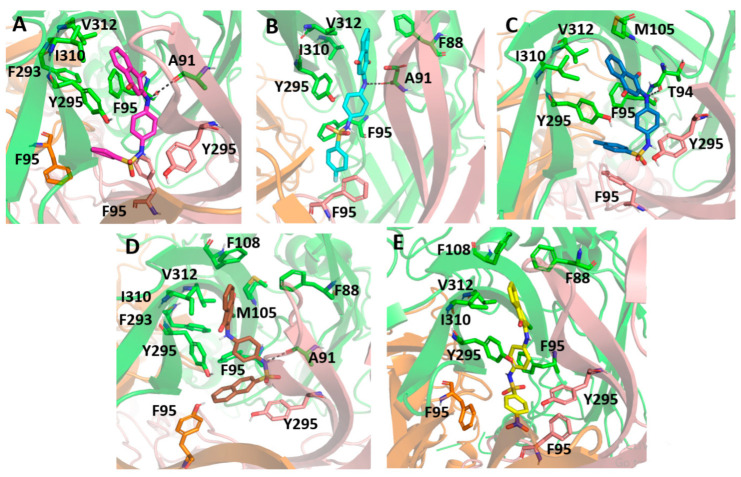
Molecular docking of the sulfonamide derivatives into the P2X7 allosteric pocket. The P2X7R is depicted in cartoon and the main residue involved in the interaction is depicted in stick (**A**) In pink is represented the PS01 (**B**) In cyan is represented the PS02 (**C**) In dark blue is represented the PS03 (**D**) In brown is represented the PS09 (**E**) In yellow is represented the PS10.

Figure 9 presents the sulfonamide compound interactions extracted from the last frame of the molecular dynamics. It is important to note that the addition of a small hydrophobic group at the para position on the benzenesulfonamide could contribute to a favorable binding affinity, since the compounds could move deeper into the cavity and perform hydrophobic interactions with residues located in deeper positions, such as Phe95, from the three different subunits, and Tyr295. On the other hand, compound PS09 presented an extra ring at this position that prevented it from performing such an interaction. Additionally, the flourination at this site, e.g., in PS02, was an interesting approach, not only for the hydrophobic contribution, but also for preventing the metabolic reaction of fase I at this site. Compound PS10 presented a NO_2_ moiety at this site, and the electron-withdrawing effect caused by the nitro group in the aromatic ring changed the polarity of the compound, reducing the negative quadrupole of the aromatic ring. This fact favored parallel π-stacking conformations with the lateral chain from Tyr295.

**Figure 10:**
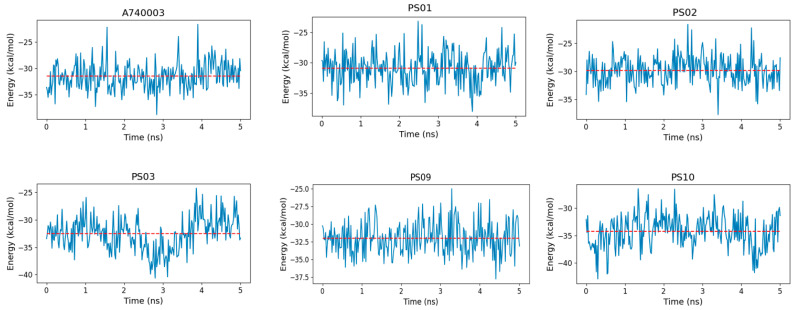
Binding free energy fluctuation of the ligands (A740003, PS01, PS02, PS03, PS09, and PS10) into the P2X7 allosteric binding site during the last 5 ns of simulation for each complex trajectory. The blue line indicates the binding free energy fluctuation, and the dashed red line indicates the binding free energy average.

The binding free energy indicated that sulfonamides had an affinity for the P2X7 receptor comparable to that of the known inhibitor A740003 (Figure 10). Moreover, PS10 presented a slightly more favorable binding free energy than A740003, which might be due to PS10 being more buried inside the P2X7 pore, which allowed this ligand to carry out hydrophobic interactions with residues from the three subunits located deep in the allosteric site. In addition, a strong staggered π-stacking interaction formed among Tyr295, the PS10 phenyl moiety and Phe95, stabilizing the PS10 conformation and contributing to a low binding free energy.

## 3. Experimental Section

### 3.1. Chemistry

The naphthoquinone sulfonamide derivatives (PS01 to PS10) and sulfonate ester derivatives (PS11 to PS15) tested in this work were synthetized as previously reported [28].

### 3.2. In Vitro Assays

#### 3.2.1. Peritoneal Macrophage Isolation and Culture

The animals used in these experiments were furnished by the animal breeding unit at Pavilion Hélio and Peggy Pereira at FIOCRUZ/IOC. The Institutional Animal Ethics Committee approved all experimental protocols under number L039-2016. Peritoneal macrophages were isolated from euthanized mice (weighing between 20 and 30 g) by injection of 10 mL phosphate-buffered saline (PBS) into the peritoneum of each animal. Then, the solution was completely removed from the peritoneal cavity using a syringe and transferred to 50 mL sterile tubes. Tubes were centrifuged at 1500 rpm for 5 min. The supernatant was discarded, and the pellet was resuspended in RPMI medium supplemented with 10% SFB and 1% antibiotic. The cells were counted and seeded in plates. After 40 min, the supernatant from each well was discarded to remove cells other than macrophages, and then RPMI medium was added. The macrophages were maintained in RPMI medium supplemented with 10% SFB and 1% antibiotic in a humid atmosphere at 37 °C containing 5% CO_2_ during the experiments.

#### 3.2.2. Resazurin Reduction Assay

For cell viability estimation, the resazurin reduction assay, commercially known as Alamar blue [39], was used. Briefly, the cells were seeded in 96-well plates at a concentration of 3.0 × 10^5^ cells/well and kept in an oven at 37 °C under a 5% CO_2_ atmosphere. Then, the cultures were treated with 10 μM of each naphthoquinone derivative for 24 h. To reveal the assay, the treated cells were incubated for 4 h with resazurin, and the fluorescence of the material was determined using a SpectraMax M5 spectrophotometer (Molecular Devices, San Jose, CA, USA) at wavelengths of 570/595 nm (excitation/emission). For the positive control of the reaction, 0.5% Triton-X 100 surfactant (Sigma-Aldrich, St. Louis, MO, USA) was used.

#### 3.2.3. LDH Release Assay

Peritoneal macrophages (3.0 × 10^5^ cells/well) were grown in 96-well plates and kept in an oven at 37 °C under a 5% CO_2_ atmosphere. The culture was treated with 10 μM of the substances for 24 h. The assay was finished by the addition of the LDH kit’s reagent (Kit CytoTox 96® Non-Radioactive Cytotoxicity Assay, Promega, Madison, WI, USA), prepared according to the manufacturer’s instructions. After 30 min of reaction, a stop solution was added, and the absorbance was measured at 490 nm.

#### 3.2.4. Dye Uptake Assay

To evaluate P2X7R-associated pore formation, a propidium iodide (PI) uptake assay was used. Peritoneal macrophages (3.0 × 10^5^/well) were incubated with naphthoquinone derivatives and the inhibitor BBG (Brilliant Blue G, Sigma-Aldrich) at 10 µM and 750 nM, respectively, for 10 min. Subsequently, cells were exposed to 5 mM ATP for 15 min, and in the last 5 min, they were incubated with 1 µM PI dye. At the end of the incubation period, fluorescence was measured using a SpectraMax M5 spectrophotometer at 493/636 nm (excitation/emission). Compounds with inhibition values higher than 50% were selected to evaluate their inhibitory activity at varying concentrations. The inhibition curve was obtained using compounds at concentrations of 0.01 µM, 1 µM, 10 µM, 50 µM, and 100 µM. The controls and the procedure were the same.

#### 3.2.5. IL-1β Enzyme-Linked Immunosorbent Assay

The monocytic THP-1 cell line used in this experiment was donated by the Laboratory of Applied Pharmacology, Institute of Drug Technology (Farmanguinhos), Rio de Janeiro, Brazil. Cells were plated at 2 × 10^5^ cells/well in 96-well culture plates maintained in RPMI containing 10% fetal bovine serum (FBS), penicillin (100 U/mL), and streptomycin (100 mg/mL) in a humidified 5% CO_2_ atmosphere at 37 °C. Then, the cells were primed with *Escherichia coli* lipopolysaccharide (LPS, serotype 0127: B8) four hours before ATP stimulation for 30 min. Mouse peritoneal macrophages were primed with 10 ng/mL interferon-γ (IFN-γ) for 24 h and posteriorly stimulated with *Escherichia coli* lipopolysaccharide (LPS, serotype 0127: B8). The P2X7R antagonist A740003 and naphthoquinone derivatives were preincubated for 30 min before ATP addition. IL-1β was determined according to a standard kit (Bioscience, San Diego, CA, USA and Abcam, Cambridge Biomedical Campus, Cambridge, UK).

### 3.3. In Vivo Assays

#### 3.3.1. ATP-Induced Paw Edema Assay

Paw edema in mice was induced by intra-plantar administration of ATP (10 mg/paw) for 30 min. Animals were pretreated intraperitoneally with the standard P2X7R antagonist A740003 (1 mg/kg) and the selected compounds PS01, PS02, PS03, PS09, and PS10 (1 mg/kg) for 60 min before ATP administration. Edema formation was determined with a mouse plethysmometer (UGO Basile, Gemonio, Italy) before ATP injection compared with saline injection in the contralateral paw edema.

#### 3.3.2. Ligands and Receptor Modeling

Initially, the compounds were built in the program Avogadro [23,39], and then the molecular geometries were optimized using the MMFF94 force field [40]. Finally, the optimized conformers were submitted to refinement calculations of the optimized geometry through the semiempirical PM6 method performed by MOPAC2016 [41,42,43]. The three-dimensional structure of the human P2X7R ion channel model was built as described in Faria et al., 2018 [38]. The primary sequence was obtained from the UniProtKB database (code: Q99572) [44]. As in this work we studied molecules with allosteric antagonist activity (PS01, PS02, PS03, PS09, and PS10), we used the crystallographic structure deposited under the PDB code of 5U1U as the template, since this structure is bound to the ligand A740003, which is a known allosteric inhibitor [37]. The ClustalW program (EMBL-EBI, Cambridgeshire, UK) was used for sequence alignment (Supplementary Materials, Figure S1) [45]. The ProsA program (Insoft Oy, Kempele, Finland) was used to assess the Z score and local model quality (Figure S3 and Figure S4), and the PROCHECK program (EMBL-EBI) was used to generate the Ramachandran plot (Figure S5) [46,47].

#### 3.3.3. Molecular Docking

The ligand and P2X7 model were previously prepared using AutoDock tools 4.2.6 (CCSB, La Jolla, CA, USA) in which hydrogen atoms were added, as well as Gasteiger partial charges [48]. As the biological analysis indicated potential allosteric antagonist activity for the molecules, we placed the grid box around the allosteric site. The allosteric site comprised a groove located in the upper body domains of the P2X7 neighboring subunits. The main residues involved in the interaction with the allosteric antagonist were Phe95, Phe103, Met105, Phe293, and V312 [37]. Molecular docking was performed using the AutoDockVina program (CCSB, La Jolla, CA, USA) [49]. The exhaustiveness parameter was set to 10, and the energy range parameter was set to 30. This molecular docking approach was previously supported by redocking with the ligand A740003, described in Faria et al., 2018 [38].

#### 3.3.4. Molecular Dynamics

Molecular dynamics was performed to evaluate the complex stability generated by the molecular docking process. The ligand force field parameters were previously generated by the Antechamber program [50]. The tLeap (Amber 18.0 Module) was used to generate the molecular system topology (ligand and protein). For the protein and ligands, the force field implemented was the FF14SB and GAFF (Generalized Amber Force Field), respectively [51]. The water model applied was TIP3P [52]. The water molecules were arranged around the solute at 1.5 Å and with a distance from the border of the simulation box of 15 Å. Additional ions (Na^+^ and Cl^−^) were added to neutralize the molecular system charges. Finally, the systems were minimized using the steepest descent method followed by the conjugate gradient method with 5000 iteration cycles. The particle-mesh–Ewald method was used for the treatment of long-range interactions, and a 9 Å limit was selected to compute the nonbonded interactions. The simulations were carried out by AMBER 18.0 with the PMEMD version (Particle Mesh Ewald Molecular Dynamics). Each complex system was thermalized with gradual heating of 30 K every 10 ps until reaching 300 K. The thermalization was performed in a canonical ensemble employing the Langevin thermostat. Next, the systems were equilibrated for 10 ps at 300 K under 1 atm pressure employing the Berendesen barostat [53]. Finally, molecular dynamics production was performed over 50 ns for each complex, with a time step for integration of 2 fs under canonical ensemble and periodic boundary conditions. The SHAKE algorithm was employed on atoms covalently bonded to a hydrogen atom. Root-mean-square deviations (RMSDs) were calculated to analyze the molecular dynamics trajectories. We performed the following two different RMSD measurements: one for the whole system (receptor and ligand) to evaluate the overall system stability, and the other for the ligand alone to evaluate the ligand trajectory and its stability in the binding pocket. However, in this case, we applied the “nofit” function to avoid superimposing the ligand structures onto each other prior to calculating RMSD, thus harnessing the movement of the inhibitors in relation to the receptor. Trajectory visual inspections were performed using the VMD program (Visual Molecular Dynamics version 1.9.2, Urbana, IL, USA).

#### 3.3.5. Binding Free Energy Determination

To evaluate the stability of each complex and compare the sulfonamide affinity with the inhibitor A740003 at the P2X7 receptor, we performed binding free energy calculations. The binding free energy was calculated over the last 5 ns of simulation for each complex system. The generalized born method was applied by the MMPBSA.py script available in the AMBER 18 package [54].

### 3.4. Statistical Analysis

PRISM^®^ software (GraphPad, Inc., San Diego, CA, USA) was used for all statistical analyses. The measurements were performed in triplicate and, at least, on three independent days. All data are presented as the mean ± standard deviation of the means. Analysis of variance (ANOVA), followed by Tukey’s test, was applied to all data. Values of *p* < 0.05 were considered statistically significant.

## 4. Conclusions

Ionotropic P2X7R has been implicated in the pathology of several inflammatory and noninflammatory diseases [55,56,57,58,59,60,61]. However, to date, no approved drugs targeting the P2X7 receptor have advanced to clinical usage, mainly due to a lack of selectivity. Our group has devoted great effort to the identification of novel chemical scaffolds to selectively target the P2X7 receptor. In recent years, we reported different groups of molecules with promising activity as P2X7 receptor inhibitors. Several synthetically modified naphthoquinones proved to be good molecular platforms for P2X7 receptor antagonists, as validated by in vitro, in vivo and in silico studies.

Herein, we reported the identification of a group of naphthoquinone derivatives with promising inhibitory activity against the P2X7 receptor. Among 15 compounds, we identified 5 naphthoquinone sulfonamide derivatives (PS01, PS02, PS03, PS09, PS10) with low toxicity against mammalian cells and better performance in the inhibition of ATP-induced dye uptake and P2X7 receptor-mediated IL-1 release than well-known P2X7 receptor antagonists. These compounds also presented promising anti-edematogenic effects of sulfonamides in an ATP-induced paw edema model. The molecular docking and dynamics results indicated that all the analyzed molecules were capable of interacting with the same residues involved with the known allosteric antagonists. The binding free energy indicated that sulfonamides have an affinity for the P2X7 receptor similar to the known inhibitor A740003. In addition, PS10 presented a more favorable binding free energy than A740003. All the results presented here support these compounds as being valuable synthetic platforms for obtaining novel and more selective P2X7R antagonists.

## Figures and Tables

**Table 1:** P2X7 receptor antagonists and the selected sulfonamides inhibited ATP−induced IL−1β release.

P2X7R Inhibitors and 6a–d	IC_50_ mP2X7R (µM) IL−1β Release	IC_50_ hP2X7R (µM) IL−1β Release
BBG	24 ± 3	16 ± 2
A740003	0.9 ± 0.1	0.85 ± 0.1
PS01	5 ± 1	4 ± 2
PS02	3 ± 0.2	3 ± 0.3
PS03	1.5 ± 0.4	1 ± 0.4
PS09	0.09 ± 0.01	0.02 ± 0.002
PS10	0.7 ± 0.1	0.3 ± 0.06

## Data Availability

The authors declare that all data supporting the findings of this study are available within the article and its Supplementary Material files.

## Supplementary Materials

The following supporting information can be downloaded at: https://www.mdpi.com/article/10.3390/molecules28020590/s1, Figure S1: RMSD variation of the ligands complexed with the P2X7 receptor during 50 ns of molecular dynamics simulation; Figure S2: Sequence alignment between the target P2X7 sequence and the template generated by the ClustalW program; Figure S3: Overall model quality of the P2X7 model generated by the ProSA; Figure S4: Local model quality of the P2X7 model generated by the ProSA; Figure S5: Ramachandran plot generated by the PROCHECK program.

## Sample Availability

Samples of the compounds are available from the authors.

## Abbreviations

ATP	Adenosine triphosphate
ATPe	Extracellular ATP
BBG	Brilliant Blue G
CINC-1	Cytokine-induced neutrophil chemoattractant-1
Da	Dalton
DMSO	Dimethyl sulfoxide
FBS	Fetal bovine serum
FP	Hybrid substances nomenclature
GPCR	G protein-coupled receptors
IL-1β	Interleukin-1β
IL-6	Interleukin-6
P2X7R	P2X7 Receptor
PBS	Phosphate-Buffered Saline
PI	Propidium iodide
TNF-α	Tumor necrosis fator alfa
Tx	Triton X-100
VEGF	Vascular endothelial growth factor

## References

[1] Burnstock G., Williams M. (2000). P2 purinergic receptors: Modulation of cell function and therapeutic potential. J. Pharmacol. Exp. Ther..

[2] Ralevic V., Burnstock G. (1998). Receptors for purines and pyrimidines. Pharmacol. Rev..

[3] Burnstock G. (2014). Purinergic signalling: From discovery to current developments. Exp. Physiol..

[4] Ijzerman A.P., Jacobson K.A., Müller C.E., Cronstein B.N., Cunha R.A. (2022). International Union of Basic and Clinical Pharmacology. CXII: Adenosine Receptors: A Further UpdateS. Pharmacol. Rev..

[5] Illes P., Müller C.E., Jacobson K.A., Grutter T., Nicke A., Fountain S.J., Kennedy C., Schmalzing G., Jarvis M.F., Stojilkovic S.S. (2020). Update of P2X receptor properties and their pharmacology: IUPHAR Review 30. Br. J. Pharmacol..

[6] von Kügelgen I. (2021). Molecular pharmacology of P2Y receptor subtypes. Biochem. Pharmacol..

[7] North R.A. (2016). P2X receptors. Phil. Trans. R. Soc. B.

[8] Di Virgilio F., Dal Ben D., Sarti A.C., Giuliani A.L., Falzoni S. (2017). The P2X7 Receptor in Infection and Inflammation. Immunity.

[9] Mehta N., Kaur M., Singh M., Chand S., Vyas B., Silakari P., Bahia M.S., Silakari O. (2014). Purinergic receptor P2X7: A novel target for anti-inflammatory therapy. Bioorganic Med. Chem..

[10] Burnstock G. (2016). P2X ion channel receptors and inflammation. Purinergic Signal..

[11] Burnstock G., Knight G.E. (2018). The potential of P2X7 receptors as a therapeutic target, including inflammation and tumour progression. Purinergic Signal..

[12] Carroll W.A., Donnelly-Roberts D., Jarvis M.F. (2009). Selective P2X7 receptor antagonists for chronic inflammation and pain. Purinergic Signal..

[13] Scarpellino G., Genova T., Munaron L. (2019). Purinergic P2X7 Receptor: A Cation Channel Sensitive to Tumor Microenvironment. Recent Pat. Anti-Cancer Drug Discov..

[14] Guerra Martinez C. (2019). P2X7 receptor in cardiovascular disease: The heart side. Clin. Exp. Pharmacol. Physiol..

[15] Chrovian C.C., Rech J.C., Bhattacharya A., Letavic M.A. (2014). P2X7 Antagonists as potential therapeutic agents for the treatment of CNS disorders. Progress in Medicinal Chemistry.

[16] Eser A., Colombel J.F., Rutgeerts P., Vermeire S., Vogelsang H., Braddock M., Persson T., Reinisch W. (2015). Safety and Efficacy of an Oral Inhibitor of the Purinergic Receptor P2X7 in Adult Patients with Moderately to Severely Active Crohn’s Disease: A Randomized Placebo-controlled, Double-blind, Phase IIa Study. Inflamm. Bowel Dis..

[17] Pevarello P., Bovolenta S., Tarroni P., Za L., Severi E., Torino D., Vitalone R. (2017). P2X7 antagonists for CNS indications: Recent patent disclosures. Pharm. Pat. Anal..

[18] Bhattacharya A., Biber K. (2016). The microglial ATP-gated ion channel P2X7 as a CNS drug target. Glia.

[19] Bartlett R., Stokes L., Sluyter R. (2014). The p2x7 receptor channel: Recent developments and the use of p2x7 antagonists in models of disease. Pharmacol. Rev..

[20] Magalhães B.Q., Machado F.P., Sanches P.S., Lima B., Falcão D.Q., Von Ranke N.L., Bello M.L., Rodrigues C.R., Santos M.G., Rocha L. (2022). Eugenia sulcata (Myrtaceae) Nanoemulsion Enhances the Inhibitory Activity of the Essential Oil on P2X7R and Inflammatory Response In Vivo. Pharmaceutics.

[21] dos Santos J.P.S., Ribeiro R.C.B., Faria J.V., Bello M.L., Lima C.G.S., Pauli F.P., Borges A.A., Rocha D.R., Moraes M.G., Forezi L.S.M. (2022). Synthesis, biological evaluation and molecular modeling studies of novel 1,2,3-triazole-linked menadione-furan derivatives as P2X7 inhibitors. J. Bioenerg. Biomembr..

[22] Arruda J.C.C., Rocha N.C., Santos E.G., Ferreira L.G.B., Bello M.L., Penido C., Costa T.E.M.M., Santos J.A.A., Ribeiro I.M., Tomassini T.C.B. (2021). Physalin pool from Physalis angulata L. leaves and physalin D inhibit P2X7 receptor function in vitro and acute lung injury in vivo. Biomed. Pharmacother..

[23] de Luna Martins D., Borges A.A., e Silva N.A.d.A., Faria J.V., Hoelz L.V.B., de Souza H.V.C.M., Bello M.L., Boechat N., Ferreira V.F., Faria R.X. (2020). P2X7 receptor inhibition by 2-amino-3-aryl-1,4-naphthoquinones. Bioorg. Chem..

[24] Gonzaga D.T., Oliveira F.H., Salles J.P., Bello M.L., Rodrigues C.R., Castro H.C., de Souza H., Reis C., Leme R., Mafra J. (2019). Synthesis, biological evaluation and molecular modeling studies of new thiadiazole derivatives as potent P2X7 receptor inhibitors. Front. Chem..

[25] Faria R.X., de Jesus Hiller N., Salles J.P., Resende J.A.L.C., Diogo R.T., von Ranke N.L., Bello M.L., Rodrigues C.R., Castro H.C., de Luna Martins D. (2019). Arylboronic acids inhibit P2X7 receptor function and the acute inflammatory response. J. Bioenerg. Biomembr..

[26] Pacheco P.A.F., Galvão R.M.S., Faria A.F.M., Von Ranke N.L., Rangel M.S., Ribeiro T.M., Bello M.L., Rodrigues C.R., Ferreira V.F., da Rocha D.R. (2019). 8-Hydroxy-2-(1H-1,2,3-triazol-1-yl)-1,4-naphtoquinone derivatives inhibited P2X7 Receptor-Induced dye uptake into murine Macrophages. Bioorganic Med. Chem..

[27] Zaid A., Gérardin P., Taylor A., Mostafavi H., Malvy D., Mahalingam S. (2018). Chikungunya Arthritis: Implications of Acute and Chronic Inflammation Mechanisms on Disease Management. Arthritis Rheumatol..

[28] Pacheco P.A.F., Gonzaga D.T.G., Cirne-Santos C.C., Barros C.S., Gomes M.W.L., Gomes R.S.P., Gonçalves M.C., Ferreira V.F., Rabelo V.W.H., Abreu P.A. (2022). Synthesis and Anti-Chikungunya Virus (CHIKV) Activity of Novel 1,4-Naphthoquinone Sulfonamide and Sulfonate Ester Derivatives. J. Braz. Chem. Soc..

[29] Adinolfi E., Callegari M.G., Ferrari D., Bolognesi C., Minelli M., Wieckowski M.R., Pinton P., Rizzuto R., Di Virgilio F. (2005). Basal activation of the P2X7 ATP receptor elevates mitochondrial calcium and potential, increases cellular ATP levels, and promotes serum-independent growth. Mol. Biol. Cell.

[30] Kan L.K., Seneviratne S., Drummond K.J., Williams D.A., O’Brien T.J., Monif M. (2020). P2X7 receptor antagonism inhibits tumour growth in human high-grade gliomas. Purinergic Signal..

[31] Pegoraro A., De Marchi E., Ferracin M., Orioli E., Zanoni M., Bassi C., Tesei A., Capece M., Dika E., Negrini M. (2021). P2X7 promotes metastatic spreading and triggers release of miRNA-containing exosomes and microvesicles from melanoma cells. Cell Death Dis..

[32] Faria R.X., DeFarias F.P., Alves L.A. (2005). Are second messengers crucial for opening the pore associated with P2X7 receptor?. Am. J. Physiol. Cell Physiol..

[33] Faria R.X., Cascabulho C.M., Reis R.A.M., Alves L.A. (2010). Large-conductance channel formation mediated by P2X7 receptor activation is regulated through distinct intracellular signaling pathways in peritoneal macrophages and 2BH4 cells. Naunyn. Schmiedebergs. Arch. Pharmacol..

[34] Jiang L.H., Mackenzie A.B., North R.A., Surprenant A. (2000). Brilliant blue G selectively blocks ATP-gated rat P2X7 receptors. Mol. Pharmacol..

[35] Katsuno K., Burrows J.N., Duncan K., Van Huijsduijnen R.H., Kaneko T., Kita K., Mowbray C.E., Schmatz D., Warner P., Slingsby B.T. (2015). Hit and lead criteria in drug discovery for infectious diseases of the developing world. Nat. Rev. Drug Discov..

[36] Koutsoni O., Karampetsou K., Dotsika E. (2019). In vitro Screening of Antileishmanial Activity of Natural Product Compounds: Determination of IC50, CC50 and SI Values. Bio-Protocol.

[37] Karasawa A., Kawate T. (2016). Structural basis for subtype-specific inhibition of the P2X7 receptor. Elife.

[38] Faria R.X., Oliveira F.H., Salles J.P., Oliveira A.S., von Ranke N.L., Bello M.L., Rodrigues C.R., Castro H.C., Louvis A.R., Martins D.L. (2018). 1,4-Naphthoquinones potently inhibiting P2X7 receptor activity. Eur. J. Med. Chem..

[39] O’Brien J., Wilson I., Orton T., Pognan F. (2000). Investigation of the Alamar Blue (resazurin) fluorescent dye for the assessment of mammalian cell cytotoxicity. Eur J Biochem..

[40] Hanwell M.D., Curtis D.E., Lonie D.C., Vandermeerschd T., Zurek E., Hutchison G.R. (2012). Avogadro: An advanced semantic chemical editor, visualization, and analysis platform. J. Cheminform..

[41] Halgren T.A. (1996). Merck molecular force field. I. Basis, form, scope, parameterization, and performance of MMFF94. J. Comput. Chem..

[42] Stewart J.J.P. (2007). Optimization of parameters for semiempirical methods V: Modification of NDDO approximations and application to 70 elements. J. Mol. Model..

[43] Stewart J.J.P. (2013). Optimization of parameters for semiempirical methods VI: More modifications to the NDDO approximations and re-optimization of parameters. J. Mol. Model..

[44] Stewart J.J.P. Stewart computational chemistry. http://openmopac.net.

[45] Bateman A., Martin M.J., Orchard S., Magrane M., Agivetova R., Ahmad S., Alpi E., Bowler-Barnett E.H., Britto R., Bursteinas B. (2021). UniProt: The universal protein knowledgebase in 2021. Nucleic Acids Res..

[46] Thompson J.D., Higgins D.G., Gibson T.J. (1994). CLUSTAL W: Improving the sensitivity of progressive multiple sequence alignment through sequence weighting, position-specific gap penalties and weight matrix choice. Nucleic Acids Res..

[47] Laskowski R.A., MacArthur M.W., Moss D.S., Thornton J.M. (1993). PROCHECK: A program to check the stereochemical quality of protein structures. J. Appl. Crystallogr..

[48] Yang J., Zhang Y. (2015). I-TASSER server: New development for protein structure and function predictions. Nucleic Acids Res..

[49] Goodsell D.S., Olson A.J. (1990). Automated docking of substrates to proteins by simulated annealing. Proteins Struct. Funct. Bioinforma..

[50] Trott O., Olson A.J. (2009). AutoDock Vina: Improving the speed and accuracy of docking with a new scoring function, efficient optimization, and multithreading. J. Comput. Chem..

[51] Wang J., Wolf R.M., Caldwell J.W., Kollman P.A., Case D.A. (2004). Development and testing of a general Amber force field. J. Comput. Chem..

[52] Jorgensen W.L., Chandrasekhar J., Madura J.D., Impey R.W., Klein M.L. (1983). Comparison of simple potential functions for simulating liquid water. J. Chem. Phys..

[53] Berendsen H.J.C., Postma J.P.M., Van Gunsteren W.F., Dinola A., Haak J.R. (1984). Molecular dynamics with coupling to an external bath. J. Chem. Phys..

[54] Humphrey W., Dalke A., Schulten K. (1996). VMD: Visual molecular dynamics. J. Mol. Graph..

[55] Miller B.R., McGee T.D., Swails J.M., Homeyer N., Gohlke H., Roitberg A.E. (2012). MMPBSA.py: An efficient program for end-state free energy calculations. J. Chem. Theory Comput..

[56] Savio L.E., de Andrade Mello P., Da Silva C.G., Coutinho-Silva R. (2018). The P2X7 receptor in inflammatory diseases: Angel or demon?. Front. Pharmacol..

[57] Ren W.J., Illes P. (2022). Involvement of P2X7 receptors in chronic pain disorders. Purinergic Signal..

[58] Andrejew R., Oliveira-Giacomelli Á., Ribeiro D.E., Glaser T., Arnaud-Sampaio V.F., Lameu C., Ulrich H. (2020). The P2X7 Receptor: Central Hub of Brain Diseases. Front. Mol. Neurosci..

[59] Pacheco P.A.F., Faria R.X. (2021). The potential involvement of P2X7 receptor in COVID-19 pathogenesis: A new therapeutic target?. Scand. J. Immunol..

[60] Skaper S.D., Debetto P., Giusti P. (2010). The P2X7 purinergic receptor: From physiology to neurological disorders. FASEB J..

[61] Lara R., Adinolfi E., Harwood C.A., Philpott M., Barden J.A., Di Virgilio F., McNulty S. (2020). P2X7 in Cancer: From Molecular Mechanisms to Therapeutics. Front. Pharmacol..

