# Supplementary material for: Synthesis, Biological Evaluation and Molecular Modeling Studies of Naphthoquinone Sulfonamides and Sulfonate Ester Derivatives as P2X7 Inhibitors
Source: Molecules. 2023 Jan 6;28(2):590. doi: 10.3390/molecules28020590 (PMC9866630; doi:10.3390/molecules28020590)
Supplement: Supplementary file 1 [file molecules-28-00590-s001.zip › molecules-2085366-supplementary.pdf]

Supplementary Material

Synthesis, Biological Evaluation and Molecular Modeling Studies of Naphthoquinone Sulfonamides and Sulfonate ester Derivatives as P2X7 Inhibitors

Paulo Anastácio Furtado Pacheco [1, †], Daniel Tadeu Gomes Gonzaga [2], Natalia Lidmar von Ranke [3], Carlos Rangel Rodrigues [3], David Rodrigues da Rocha 1, Fernando de Carvalho da Silva [1], Vitor Francisco Ferreira[1] and Robson Xavier Faria [4,5,†,*]

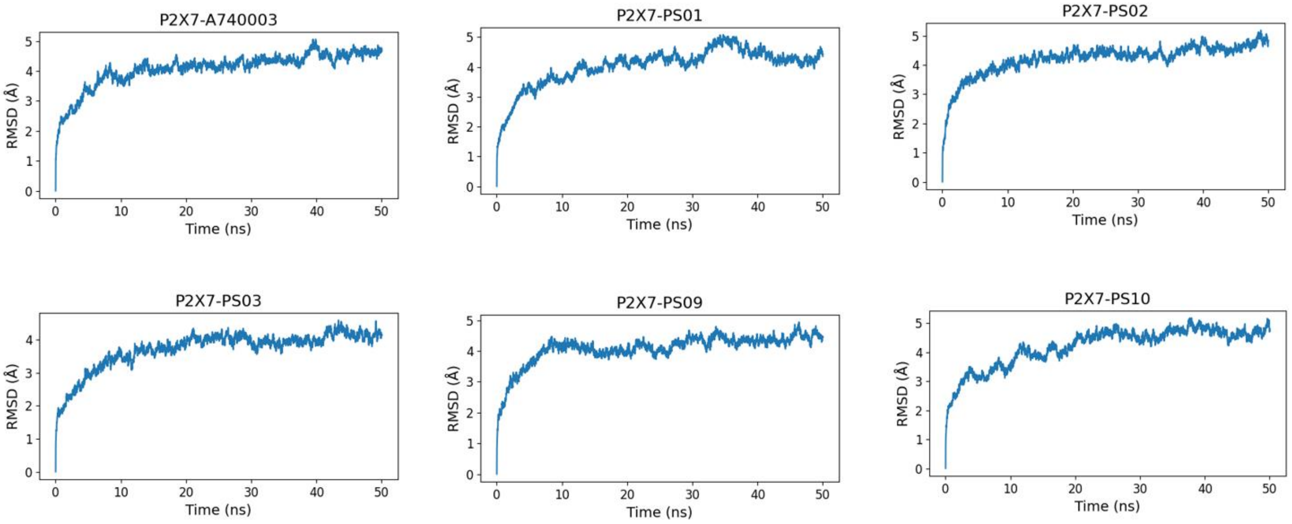

Figure S1. RMSD (Root-mean-square deviation) variation of the ligands (A740003, PS01, PS02, PS03, PS09, and PS10) complexed with the P2X7 receptor, during 50 ns of molecular dynamics simulation. The title of each plot indicates each complex RMSD variation.

```
sp|Q99572|P2RX7_HUMAN      MPACCSCSDVFQYETNKVTRIQSMNYGTIKWFFHVIIFSYVCFALVSDKL
5U1U_1|Chain               --------------------GSSTNYGTIKWIFHALVFSYISFALISDKR
                                                .* *******:**.::***:.***:*** 

sp|Q99572|P2RX7_HUMAN      YQRKEPVISSVHTKVKGIAEVKEEIVENGVKKLVHSVFDTADYTFPLQGN
5U1U_1|Chain               YQKKEPLISSVHTKVKGIAEVKAEILENGMKKMVSGVFDTADYTFPLQGN
                           **:***:*************** **:***:**:* .**************

sp|Q99572|P2RX7_HUMAN      SFFVMTNFLKTEGQEQRLCPEYPTRRTLCSSDRGCKKGWMDPQSKGIQTG
5U1U_1|Chain               SFFVMTNFIKTEGQQQGLCPDFPTARTICSSDRGCKKGRMDPQSKGIQTG
                           ********:*****:* ***::** **:********** ***********

sp|Q99572|P2RX7_HUMAN      RCVVYEGNQKTCEVSAWCPIEAVEEAPRPALLNSAENFTVLIKNNIDFPG
5U1U_1|Chain               RCVVYKERLKTCEVSAWCPIEEVKDAPRPALLNSAENFTVLIKNNIDFPG
                           *****: . ************ *::*************************

sp|Q99572|P2RX7_HUMAN      HNYTTRNILPGLNITCTFHKTQNPQCPIFRLGDIFRETGDNFSDVAIQGG
5U1U_1|Chain               HNYTTRNILPGVNITCTFHKTQNPQCPIFRLGDIFQETGDSFSDVAIQGG
                           ***********:***********************:****.*********

sp|Q99572|P2RX7_HUMAN      IMGIEIYWDCNLDRWFHHCRPKYSFRRLDDKTTNVSLYPGYNFRYAKYYK
5U1U_1|Chain               IMGIEIYWDCNLDGWFHHCRPKYSFRRLDDKTTSESLYPGYNFRYAKYYK
                           ************* *******************. ***************

sp|Q99572|P2RX7_HUMAN      ENNVEKRTLIKVFGIRFDILVFGTGGKFDIIQLVVYIGSTLSYFGLAAVF
5U1U_1|Chain               ENNVEKRTLIKVFGIRFDILVFGTGGKFNVIQLAVYIGSVISYFGLATVF
                           ****************************::***.*****.:******:**

sp|Q99572|P2RX7_HUMAN      IDFLIDTYSSNCCRSHIYPWCKCCQPCVVNEYYYRKKCESIVEPKPTLKY
5U1U_1|Chain               IDILINTYSASS--------------------------------------
                           **:**:***:..
```

Figure S2. Sequence alignment between the target P2X7 sequence (UniProtKB code: Q99572) and the template (PDB ID: 5U1U) generated by the ClustalW program.

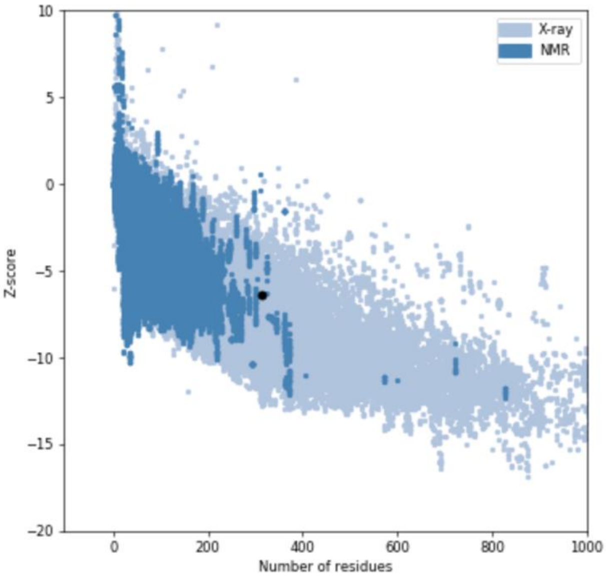

Figure S3. Overall model quality of the P2X7 model generated by the ProSA (Protein Structure Analysis). The model presented z-score value of -6.42, represented by the black spot. The blue spots represent the z-scores of all experimentally determined protein chains in current PDB. Groups of structures from different sources (NMR, X-ray) are distinguished by dark and light blue colors.

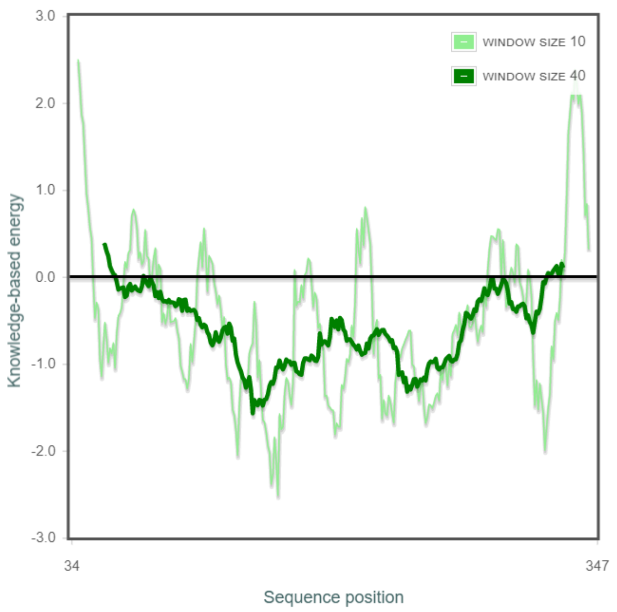

Figure S4. Local model quality of the P2X7 model generated by the ProSA (Protein Structure Analysis). The plot shows the energies as a function of amino acid sequence position i. In general, positive values correspond to problematic or erroneous parts of the input structure. A plot of single residue energies usually contains large fluctuations and is of limited value for model evaluation. Hence, the plot is smoothed by calculating the average energy over each 40-residue fragment s(i,i+39), which is then assigned to the 'central' residue of the fragment at position i+19 (thick line). A second line with a smaller window size of 10 residues is shown in the background of the plot (thin line).

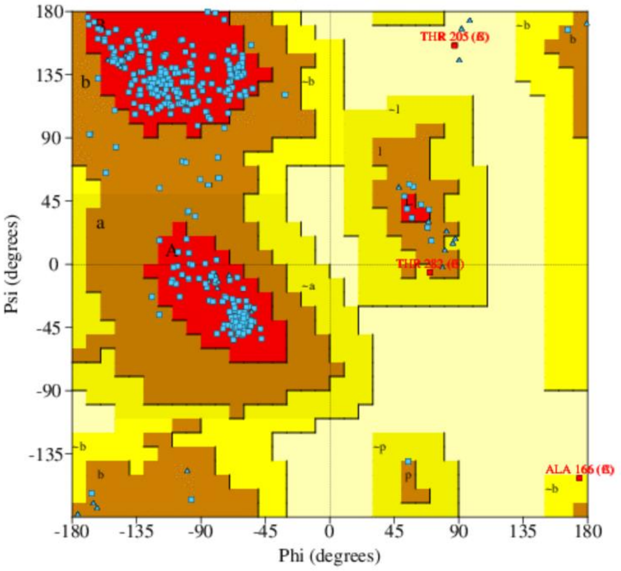

Figure S5. Ramachandran plot generated by the PROCHECK program. In this plot 91.3% of the residues were in the most favored regions, 7.6% were in additional allowed regions, 0.7% were in generously allowed regions, and only 0.4% were in disallowed regions.
